# Editorial: Tropical Montane Forests in a Changing Environment

**DOI:** 10.3389/fpls.2021.712748

**Published:** 2021-08-11

**Authors:** Norma Salinas, Eric G. Cosio, Miles Silman, Patrick Meir, Andrew T. Nottingham, Rosa Maria Roman-Cuesta, Yadvinder Malhi

**Affiliations:** ^1^Institute for Nature, Earth and Energy, Pontifical Catholic University of Peru, Lima, Peru; ^2^Chemistry Section, Pontifical Catholic University of Peru, Lima, Peru; ^3^Center for Energy, Environment, and Sustainability, Wake Forest University, Winston-Salem, NC, United States; ^4^Research School of Biology, The Australian National University, Canberra, ACT, Australia; ^5^School of Geography, University of Leeds, Leeds, United Kingdom; ^6^Laboratory of GeoInformation Science and Remote Sensing, Wageningen University and Research, Wageningen, Netherlands; ^7^Centre for International Forestry Research (CIFOR), Bogor, Indonesia; ^8^Environmental Change Institute, School of Geography and the Environment, University of Oxford, Oxford, United Kingdom

**Keywords:** carbon dynamics, cloud forests, global change, leaf traits, soil microbiome, human impact, resilience, abiotic stress

Tropical montane forests (TMFs) are found on most of Earth's continents along variable elevation ranges, whose potential upper limits are influenced by cloud condensation heights and minimum temperatures. They are most widespread in South America and in (semi-)humid mountain areas (Richter, 2008). According to FAO and UNEP (2020), the area covered by tropical and subtropical montane forests is around 305 million hectares, about 13% of the area covered by tropical and subtropical forests. Their elevational limits are difficult to establish due to the interactions of the different factors that determine their characteristics. Among these, geomorphology plays a leading role in regulating TMF structure, and provides useful clues on the contributing mechanisms. Most TMFs occur under highly variable topography, including steep slopes (Asner et al., 2014) and landslide-prone terrain (Shreve, 1914; Larsen and Torres-Sánchez, 1998). Also, the latitudinal gradient, orography, and vertical thermal gradients have a direct influence on the fauna and flora of TMFs. The latitudinal pattern is not the same in all TMFs, the temperature and precipitation conditions occur due to seasonality in the climate and are unambiguously linked to species climatic affinity preferences (Ohsawa, 1991; Cuesta et al., 2016; Chu et al., 2019). Another factor is the annual precipitation that generally exceeds c. 1,000–1,200 mm and can be associated with low level cloud cover or mist, which results in a lower incidence of sunlight and lower primary productivity, suggesting that NPP for these forests is driven by changes in photosynthesis. This highlights the importance of variations in solar radiation. Girardin et al. (2010) estimated that NPP values recorded in TMFs range widely between 4 and 7 Mg C ha^−1^ yr^−1^. Despite this variability, TMFs store significant amounts of carbon in their soils. Malhi et al. (2017) showed that the soil organic layer depth sharply increased with lower mean annual temperatures. Lower temperatures also result in low nutrient inputs through slow mineralization of organic matter (Townsend et al., 1995). Declining temperature appears to be the principal rate-limiting factor for litter decay with increasing elevation on tropical mountains (Schuur, 2001; Salinas et al., 2011). The low temperatures have also been linked to biogeochemical limitations, by reducing nitrogen availability (Nottingham et al., 2015, 2018b) and N_2_ fixation (Houlton et al., 2008). However, biogeochemical cycling in TMFs is further affected—returning to our primary driver—by geomorphology via landslide activity and uplift, which increases the supply of rock-derived nutrients such as phosphorus (Tanner et al., 1992; van de Weg et al., 2012; Nottingham et al., 2015). The novel environments TMFs represent are, thus, a product of interconnected geological and climatic forces.

Current global climate models (GCMs) suggest enhanced warming of the tropical mid and upper troposphere (Fu et al., 2011). Consequently, rates of temperature rise are expected to be larger at higher than at lower elevations, as it has already been reported in mountains around the world (Bradley et al., 2006; Roman-Cuesta et al., 2014). There is still uncertainty on the effects that temperature and changing moisture conditions will have on the cloud belt formation in TMFs (Lawton et al., 2001) but upper displacements of the condensation belt are expected (Foster, 2001; Halladay et al., 2012). Moreover, mountain regions are more frequently suffering the impacts of oceanic warming such as El Nino Southern Oscillation ENSO and North Atlantic Oscillation (NAO)/Atlantic Multidecadal Oscillation (AMO), but the effect of their drought and flooding spells on TMFs' flora and fauna are yet under-researched (Foster, 2001; Roman-Cuesta et al., 2014; Oliveras et al., 2017).

Tropical montane forest ecosystems are fragile but exceedingly valuable ecosystems, due to their important role in the provision of ecosystem services, including the regulation of water and the regional climate (Bubb et al., 2004), the capture and storage of carbon (Cuesta et al., 2009; Tejedor Garavito et al., 2012) and—not least—by harboring a vast store of biodiversity (Myers, 1995). The complex spatial and environmental gradients typical of TMFs generate a high diversity of habitats. TMFs are considered among the most biologically diverse and richest ecosystems on Earth (Kessler and Kluge, 2008; Richter, 2008), and recognized as hotspots of species endemism (Gentry, 1993).

These vital ecosystems services are under threat, as climate change is undoubtedly affecting their species composition and metabolic profiles in a myriad of ways. Along an elevation gradient, as global average temperatures rise, elevational shifts in the distribution of species toward regions of lower temperature are to be expected. A major concern is that the speed of climate change appears to be greater than the response capacity (adaptation and migration) for a large number of species in the Andean Amazon. On the one hand, adaptation within species or communities may result in the increased dominance of individuals more tolerant of change. On the other hand, species extinction may occur alongside migration and geographic displacement of susceptible populations toward areas with a more appropriate climate. This implies a high probability of extinction for plant species without this response capacity, which in turn would lead to changes in the carbon cycle, in the dynamics of ecosystems and uncertain impacts on wildlife. But these changes are evidenced not only in animals and plants, soil bacterial, and fungal communities on tropical mountains are also sensitive to temperature (Looby et al., 2016; Nottingham et al., 2018a) and may be affected by rapid climate warming with negative implications for carbon storage (Nottingham et al., 2019) and for plant species composition (Corrales et al., 2016). At the same time, these ecosystems are in a state of global threat due to the dynamics of change in land cover and use (Webster, 1995; Bubb et al., 2004). In many regions, land use patterns have created a mosaic of habitats transformed through the expansion of human activities. These fragmented forests should receive more attention when designing conservation policies. For all these reasons climate change can have severe impacts on montane tropical ecosystems by generating changes in the life zones, increasing the vulnerability of forests to fires, pests, invasive species, and greater deforestation pressure due to the establishment of productive systems with intensive management (Serreze, 2009).

The articles in this special issue aim to fill some of the existing gaps in our knowledge. These studies were conducted in a wide range of TMFs from pristine forests in protected areas to those with varying degrees of human disturbance along South America, Africa, and Asia. These studies have examined TMF responses to environmental cues in forest plots using a variety of tools which include remote sensing, on-site instrumentation, biometrics, and allometry, among others, to model field data and provide us with:

Two contributions to this special issue used leaf chemistry and traits analysis to determine if plant species are sensitive to changes in environmental conditions. Martin et al. assessed differences in 19 foliar traits in paired sun and shade leaves along a humid tropical forest elevation gradient in Peru, to determine if foliar chemical traits, such as photosynthetic pigments, and other leaf traits like LMA differ between them, and if the sources for these variations are environmental or genetic. They found that for most traits (i.e., N, foliar nutrients or defense compounds), there was no significant difference between sun and shade leaves. Other traits for growth, such as LMA and δ13C concentrations, maintain constant offsets, suggesting that the characteristics of shade leaves can be derived from those measured in sun leaves. Their findings also indicate that variation in sunlit canopy foliar traits are controlled primarily by changes in community composition, and secondarily by environmental factors, like elevation or substrate. They conclude that there are significant differences in light-sensitive traits between sun and leaves evaluated, that were maintained across a wide variety of environmental conditions along a 3,500-m elevation gradient suggesting that plasticity associated with light availability is an adaptive change. In contrast, they did not find sun-shade differences in other foliar traits related to defense and metabolism. Gong et al. evaluated the protective function and phylogenetic relationships of the transient red coloration of young leaves in some tropical plant species. They investigated the metabolism, photosynthetic activities, and chemical defenses of leaves from 250 tropical plant species with either red or green young leaves, in a tropical region of southwest China. They found that the occurrence of transient reddening of juvenile leaves in the tropics was coupled with increased levels of both anthocyanins and tannins and that the red coloration protects them from insect herbivory primarily through chemical defense. Also, the red coloration in young leaves is predominantly a result of adaptation to special tropical environmental conditions but without a significant intrinsic phylogenetic relationship between plant species and suggested that the anthocyanins might not function as light attenuators to protect for effects of high light intensity.

Two studies in this collection illustrate photosynthetic plant function related to light and leaf nutrients. Feeley et al. assessed the maximum photosynthetic thermal tolerances of more than 550 individuals of 164 tropical canopy tree species growing in a steep elevation gradient ascending from near sea level to near tree line in the northern Andes mountains of Colombia. They analyzed changes in plant thermal tolerances between elevations at the species and community level and tested the relationship between species' thermal tolerances and their changes in abundance through time in 10 forest inventory plots. They found a high amount of variation in the maximum thermal tolerance (T 50) among species co-occurring within each plot and that this tolerance decreases with plot elevation. However, their results also indicate that the relationship between T 50 and temperature is weak and extremely shallow. Ziegler et al. investigated physiological, chemical, and structural properties of leaves in mature individuals belonging to 12 tree species in a tropical montane rainforest in Rwanda, Central Africa. In this study, they explored the relative importance of area-based total leaf N content and within-leaf N allocation to photosynthetic capacity vs. light-harvesting in controlling the variation in photosynthetic capacity to explore the controls of interspecific variation in photosynthetic capacity and other leaf gas exchange traits. They found that photosynthetic capacity at a common leaf temperature of 25°C was higher in early succession species than in late. However, total leaf N content did not significantly differ between successional groups and there was no significant trade-off between relative leaf N investments in compounds maximizing photosynthetic capacity vs. compounds maximizing light harvesting.

Litton et al. provide information complementary other studies in this issue by examining how litterfall, live foliar nutrient concentration, foliar nutrient resorption efficiency, nutrient return via litterfall, and nutrient use e?ciency vary with mean annual temperature in two dominant tree species in a gradient in Hawaii. Their aim was to understand how increasing mean annual temperature impacts on the availability and ecological stoichiometry of macro and micronutrients. Their findings provide strong evidence that increased mean annual temperature alters the cycling and availability of a broad suite of nutrients in TMFs, with important implications for nutrient limitation to ecosystem processes in a warming world.

de la Cruz-Amo et al. explore the role of Andean TMFs as carbon reservoirs. They calculated the amount of carbon in aboveground and belowground carbon stocks, and in soil organic matter, along two elevation gradients in the southeast of Ecuador and North-Central Peru. They assess how carbon stocks vary along elevation gradients and determine the influence of climate, particularly precipitation seasonality, on the distribution of these stocks across different forest compartments. They report that the combination of annual mean temperature and precipitation seasonality explains the differences in mean total carbon stocks in these three compartments and also show different partitioning patterns along the elevation gradients both in Ecuador and Peru but that total carbon stocks do not change with elevation in either site.

Two studies report results analyzing the literature. Soh et al. systematically mapped all research on the effects of habitat degradation in TMFs globally to identify deficiencies in current knowledge and to guide future research prioritization. After a comprehensive review, they show that habitat degradation in TMFs impacts biodiversity at all ecological levels and is compounded by climate change. However, despite montane species being perceived as more extinction-prone, there are some indications of biotic resilience if disturbance in TMFs is less severe. They confirm that TMFs also provide important ecosystem services being the most important water provision, but that in recent years these ecosystems have come under human pressure manifested in the form of highest rates of deforestation. They highlight the poor research representation of Asian and African TMFs and list the top research priorities which, if addressed, would advance the goals of biodiversity conservation and sustainable use of resources in TMFs.

Tito et al. present an analysis of the natural variation of abiotic and biotic factors along mountain elevation gradients based on two papers that used field experiments conducted along an elevation gradient in the Peruvian tropical Andes. They highlight the potential for use of field experiments in future studies focused on determining the direct and indirect e?ects of climate change. They conclude that, despite abundant research on the effects of global change climate on TMFs, fundamental questions remain unanswered and it will be necessary to apply more fine-scale experimental approaches to help better predict future abundance and distribution patterns of species under altered climate scenarios, and that natural gradients are essential to quickly gain a more complete understanding on the possible impacts of climate change.

To date, there have been few experimental studies using artificial material to explore predation conduct. Murray et al. carried out predation experiments in Peninsular Malaysia at a landscape scale and across a suite of sites of varying disturbance. They used four di?erent prey items—artificial nests, artificial seeds, caterpillar models, and frog models—along a disturbance gradient, from pristine forests to tea plantations. Their purpose was to assess whether predation probability in different habitat types di?ers between mountain ranges, if this probability consistently varies in di?erent habitat types and if predation can be explained by vegetation structure. Their results show that they confirm the first and third hypotheses, but there is no clear trend in predation probability along a habitat disturbance gradient.

Results from this collection of articles show that responses to Global Change can vary greatly among species, ecosystems, and even microsites in TMFs. This suggests that the fate of these forests in response to climate change and greater deforestation pressure due to the establishment of productive agricultural systems with intensive management, can have severe impacts on montane tropical ecosystems by generating changes in vegetation life zones, increasing their vulnerability to fires, pests, invasive species, and having dramatic consequences on downstream ecosystem services. We hope that this selection of papers will stimulate interest and research into those wonderful ecosystems that are TMFs and their elevation gradients, and provide insights into the future of TMF ecosystems in a century of rapid climate change.

## Author Contributions

NS drafted the first version of the editorial. EC, MS, PM, AN, RR-C, and YM made edits, additions, and revisions. All authors contributed to the article and approved the submitted version.

## Author Disclaimer

Any use of trade, firm, or product names is for descriptive purposes only and does not imply endorsement by the U.S. Government.

## Conflict of Interest

The authors declare that the research was conducted in the absence of any commercial or financial relationships that could be construed as a potential conflict of interest.

## Publisher's Note

All claims expressed in this article are solely those of the authors and do not necessarily represent those of their affiliated organizations, or those of the publisher, the editors and the reviewers. Any product that may be evaluated in this article, or claim that may be made by its manufacturer, is not guaranteed or endorsed by the publisher.
